# A Population Model of Integrative Cardiovascular Physiology

**DOI:** 10.1371/journal.pone.0074329

**Published:** 2013-09-13

**Authors:** William A. Pruett, Leland D. Husband, Graham Husband, Muhammad Dakhlalla, Kyle Bellamy, Thomas G. Coleman, Robert L. Hester

**Affiliations:** Department of Physiology and Biophysics, University of Mississippi Medical Center, Jackson, Mississippi, United States of America; Université de Montréal, Canada

## Abstract

We present a small integrative model of human cardiovascular physiology. The model is population-based; rather than using best fit parameter values, we used a variant of the Metropolis algorithm to produce distributions for the parameters most associated with model sensitivity. The population is built by sampling from these distributions to create the model coefficients. The resulting models were then subjected to a hemorrhage. The population was separated into those that lost less than 15 mmHg arterial pressure (compensators), and those that lost more (decompensators). The populations were parametrically analyzed to determine baseline conditions correlating with compensation and decompensation. Analysis included single variable correlation, graphical time series analysis, and support vector machine (SVM) classification. Most variables were seen to correlate with propensity for circulatory collapse, but not sufficiently to effect reasonable classification by any single variable. Time series analysis indicated a single significant measure, the stressed blood volume, as predicting collapse *in situ*, but measurement of this quantity is clinically impossible. SVM uncovered a collection of variables and parameters that, when taken together, provided useful rubrics for classification. Due to the probabilistic origins of the method, multiple classifications were attempted, resulting in an average of 3.5 variables necessary to construct classification. The most common variables used were systemic compliance, baseline baroreceptor signal strength and total peripheral resistance, providing predictive ability exceeding 90%. The methods presented are suitable for use in any deterministic mathematical model.

## Introduction

Mathematical models are used in physiology to quantitatively describe interacting mechanisms and processes. Models help bridge the gap between our understanding of simple relationships between quantities and the complex dynamic relationships observed in the laboratory. As a minimum requirement for validity, a model must be able to replicate some part of the dynamics associated with experiment. Even this minimal requirement is naïve: in many experiments, a spectrum of responses can be obtained from a seemingly homogeneous population in response to a given perturbation.

As an example of this, consider the incidence of presyncope/syncope in humans following moderate hemorrhage: in experimental studies, around 70% of individuals exhibit signs of circulatory shock following such a hemorrhage, while 30% do not [1]–[5]. Given a deterministic model of human circulation, which outcome should be favored for establishing model parameters or determining model validity? In fact, neither outcome is preferable because neither outcome describes the true response. The current philosophy of parameterization, Best Fit Parameterization (BFP), explicitly chooses a single parameter value to define the model, which does not help solve the problem of dichotomous response.

Inter-subject variability occurs in parameters related to real anatomic or genetic details that vary from person to person, while the underlying physics and chemistry remains the same. In model terms, model coefficients (parameters) differ between subjects while the equations stay the same. We take this to imply that establishing distributions for these parameters allows the creation of a cohort of model analogous to a cohort of patients. Nonlinearities in a complex model magnify some small differences and annihilate others, allowing radically different behavior to develop in response to a perturbation in individuals that appeared similar in other conditions. Utilizing different parameter sets to generate multiple deterministic models can obviate the problem of selecting the goal outcome by producing a multitude of distinct outcomes. This transforms the problem of finding model parameters into the problem of estimating the distributions that these parameters might be drawn from. Moreover, using distributed parameters allows sophisticated classification machinery to be used on a population to determine before a perturbation is induced which individuals will respond in a particular way. The use of distributed parameters introduces two new challenges to the modeling process: the derivation of distributions that can create such a cohort, and the means to sample from such a complex entity.

The objective of this study is to test the hypothesis that a simple model of the circulatory system can produce a cohort of models capable of demonstrating the spectrum of human pressure response to hemorrhage. This would be accomplished by sampling from the jointly distributed sensitive parameters and subjecting each model to the same stimulus. As we utilize well-understood sensitivity analysis methods and classification algorithms, in this paper we focus on a description of the calibration and sampling problems. Given a statistical distribution of an output variable or variables, the goal is to assign joint distributions for the input parameters that yield the given output when sampled as a cohort. In this case we use cardiac output (CO) and total peripheral resistance (TPR) in a joint distribution. Calibration has been studied in several contexts previously: time series analysis of populations [6], [7] and numerous physiologically based pharmokinetic models [8], [9]. Methodologies have ranged from defining a parameter distribution by fiat to Bayesian calibration methods. In this case, model complexity and a lack of previous attention to likelihood functions in the general physiology literature make full-scale Bayesian analysis impossible. Instead, we calibrated the parameter distributions with an altered form of the Metropolis algorithm. This algorithm has the benefit of simultaneously solving the calibration and sampling problems.

In this paper, we describe a model of cardiovascular physiology and the implementation of the calibration algorithm. We compare the model’s mean arterial pressure (MAP), cardiac output (CO), and total peripheral resistance (TPR) response with that observed by Skillman [5]. We produce single variable correlations with the binary outcomes (compensate/decompensate), and use machine learning algorithms [10] to extract rubrics from the data for predicting circulatory failure that are minimal with respect to the number of parameters or variables that must be monitored. We emphasize tractable solutions to the problem of interpreting complex model output in a physiologically relevant manner.

## Methods

### Model overview

The model is a phenomenological description of various components in the feedback system regulating human cardiovascular physiology, and is not intended to be as complete and mechanistic as other published models [11], [12]. Rather, we intend to provide a clear canvas suitable for understanding the methodology of converting a deterministic model to a population model, and for demonstrating a scheme for analyzing the effects of parameter variation on a single outcome. The model is described by 15 equations solved as a state automata with adaptive step size. It is composed of 4 modules: blood flow autoregulation, baroreceptor reflex, renal control of fluid homeostasis, and cardiac integration. It was programmed in the HumMod schema and solved using the Model_Solver software developed by Tom Coleman and available for download at www.HumMod.org. The model and its accompanying files can be downloaded at https://github.com/HumMod/small-stoch-model.

**Figure 1:**
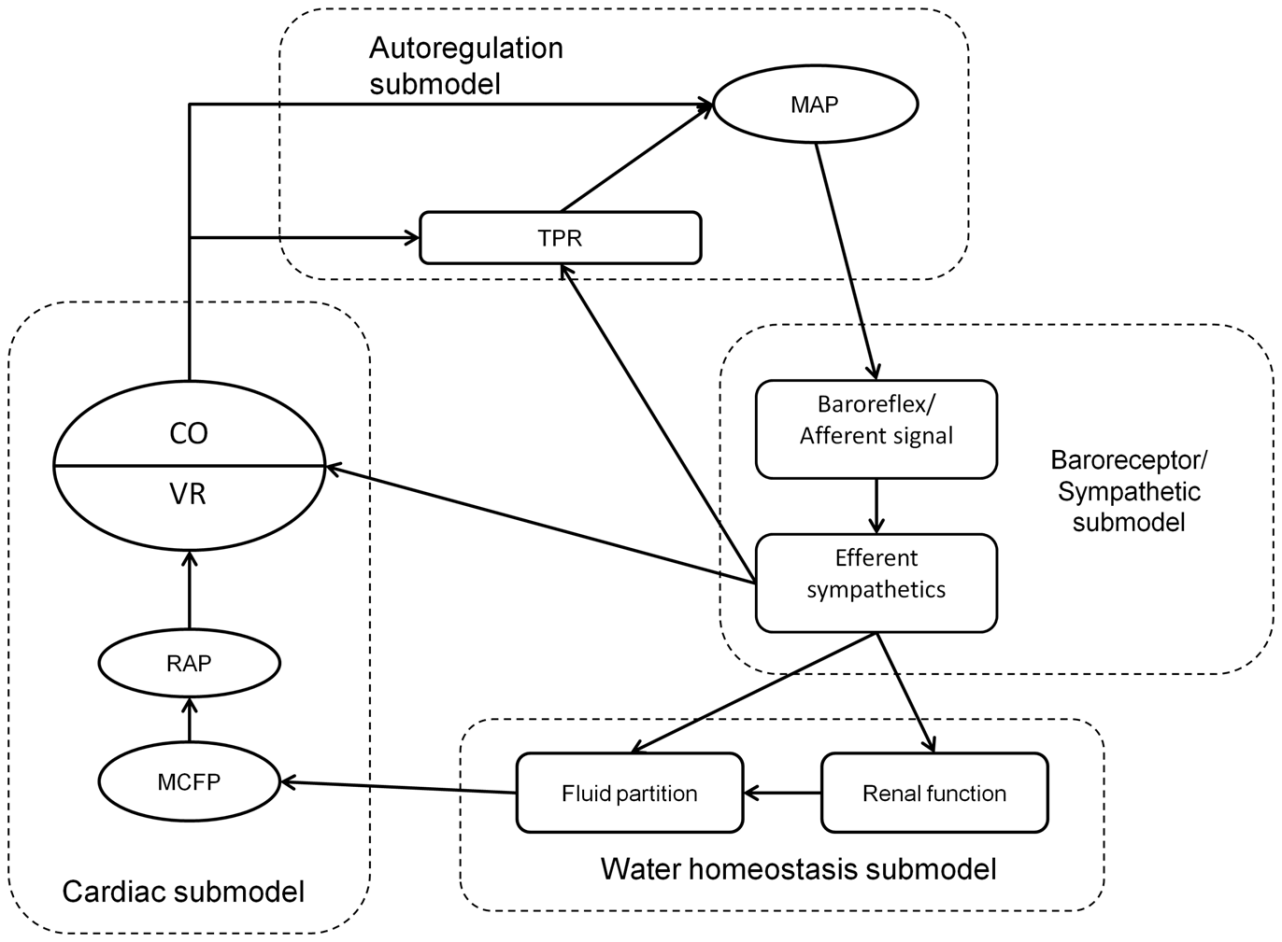
Schematic of interactions between the submodels. Ovals represent model variables, and rounded dashed boxes the submodels. The unlabeled submodel (light dashed line) is the cardiac integration model.

The conservation axiom that defines the model is the assumption that cardiac output and venous return must agree. Because both peripheral and cardiac factors can have an immediate effect on cardiac output [13]–[16], solving the problem of matching inflow and outflow is technically demanding. This model performs the task by proposing a tentative value of cardiac output and projecting its effects on peripheral components such as sympathetic activity [17], [18], flow autoregulation [15], [16], the partition of extracellular water into interstitial and blood compartments [19], and the actions of the kidney to control blood volume [20]. These peripheral factors influence right atrial pressure and thus venous return (Figure 1). By equating venous return and cardiac output and noting that venous return is bounded above and below, the resulting system can be solved uniquely as a fixed-point problem by iteration.

### Submodel: Autoregulation and mean arterial pressure

Autoregulation allows the body to maintain near constant blood flow to tissues despite changes in blood pressure [21]. In the model, cardiac output (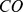) modifies peripheral resistance (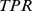) to affect mean arterial pressure (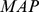); as 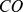 increases, so does 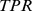 ensuring a negative feedback on 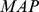. The effect on resistance is implemented via a delay with time constant 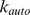; the model outputs are not sensitive to the choice of 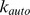 at steady state or in response to hemorrhage. We treat autoregulation throughout the body using a uniform strength and speed of response; this is a simplification for the sake of clarity. This analysis yields a functional expression for 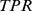:
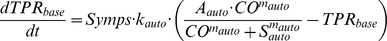



This curve is sigmoidal, with half-maximal response at the set point 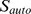 with sensitivity 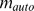. Here, 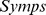 is a function defined below that simulates sympathetic outflow, and 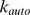 is a delay constant that controls the rate of adjustment. The same format is repeated below in other submodels. Parameter values are given in Table 1. 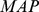 is then defined as the product of 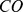 and 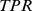,
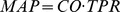



**Table 1:** Parameters of autoregulation.

Parameter	Initial Value	Description
*A_auto_*	0.0453 *mmHg*⋅*min*⋅*mL* ^−1^	Maximal autoregulation
*m_auto_*	10.86 (none)	Sensitivity of autoregulation
*S_auto_*	5126 (*mL*⋅*min* ^−1^)	Set point of autoregulation
*k_auto_*	0.00048 (none)	Delay constant of autoregulation

### Submodel: baroreceptor reflex

The baroreceptor reflex is an adaptive mechanism that buffers changes in blood pressure by increasing sympathetic outflow in response to falling pressure. In particular, increased sympathetic outflow induces increases in heart strength through vagal reflex [22], [23], increases in peripheral resistance and filling pressure by alterations in vascular tone [24], and decreases urine formation by renal sympathetic activation [25], [26].

The afferent arm of the baroreceptor senses the difference between current pressure (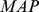) and adapted pressure (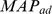). The reflex slowly adapts to new conditions, taking 1–2 days to adjust [27]. This yields the equation
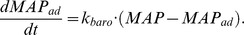



We estimate the initial value of the delay constant 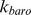 as 0.0007, corresponding to a 1 day half time for adaptation, but the probability of entering shock was sensitive to the value. For this reason, we included it as a population parameter for calibration. Given 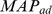, we calculate the afferent signal as
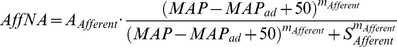



This equation is hypothesized to be sigmoidal on the domain [−50,50], corresponding to maximal effectiveness on the domain [60,160], corresponding to the range observed by Kirchheim [28]. The efferent aspect of the reflex is integrated into a single multiplier which affects each target variable (peripheral resistance, heart strength, urine output, and circulatory compliance). The value of the multiplier is
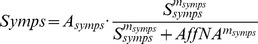



Parameter values are given in Table 2.

**Table 2:** Parameters of the baroreceptor reflex.

Parameter	Initial Value	Description
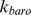	0.0007 ( 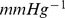 )	Delay constant of baroreceptor adaptation
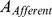	2.067 (none)	Maximal afferent outflow in response to pressure changes
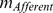	3.787 (none)	Sensitivity of afferent nerve activity response to pressure changes
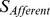	39.235 ( 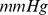 )	Set point of afferent response to pressure changes
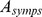	1.0041 (none)	Maximum increase in sympathetic outflow
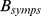	0.9333 (none)	Minimal sympathetic outflow
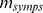	3.6046 (none)	Sensitivity of sympathetic outflow
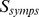	0.5349 (none)	Set point of sympathetic outflow response to afferent nerve activity

### Submodel: Renal homeostatic control of volume

To model homeostatic control of fluid volumes, we operate on the assumption that 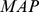 is the product of 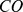 and 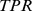, and that the kidney acts as an infinite gain feedback device to return arterial pressure to a set point defined by renal parameters and sympathetic activation [17]. Thus urine formation is a function of arterial pressure, and is reduced by increased sympathetic activity [29]. Extracellular fluid volume (ECFV) is the accumulation of fluid described by intake less excretion, represented as urine output (UO). This yields the following equations:
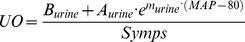


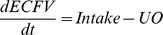



The exponential nature of the urine output equation is related to the infinite gain theory of renal regulation of volume homeostasis [30]. ECFV is partitioned into blood and interstitial volumes via the relationship
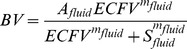



Parameter values are given in Table 3.

**Table 3:** Parameters of fluid homeostasis.

Parameter	Initial Value	Description
*Intake*	1.0 ( 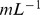 )	Water intake
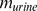	0.05 (none)	Slope constant in urine equation
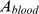	7900 ( 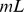 )	Maximal blood volume
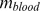	3.58 (none)	Sensitivity of blood volume to ECFV
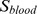	15570 ( 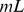 )	Set point of BV-ECFV relationship

### Submodel: Cardiac integration

With peripheral factors modeled, the model is completed by incorporating Guyton’s equations linking blood volume with circulatory and atrial pressures. This is done by noting the effects of sympathetic outflow on systemic (chiefly venous) compliance and using this value to calculate mean circulatory filling pressure (MCFP). We model these as
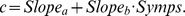


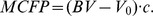



MCFP is the RAP-intercept on Guyton’s RAP-VR curve relationship [30]; the slope of the relationship is the resistance to venous return (RVR). RVR is distinct from TPR, being comprised of the arterial and venous resistances, as well as circulatory compliance. To calculate RVR, we note that the effectiveness of a vascular bed in modulating venous return through changes in resistance depends on the magnitude of the compliance upstream from the resistance. If only arterial resistance is varied while venous resistance remained fixed, about 75% of the resistance to venous return would be constant because of the dominant role of the venous compliance [30]. It is reasonable to assume that some changes in venous resistance occur with changes in total vascular resistance, leading us to postulate that about 1/2 of the resistance to venous return is fixed and 1/2 is linked to changes in total peripheral resistance. Furthermore, we assume that the relationship is linear:
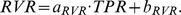



These factors can be combined to define venous return:
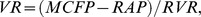
which we require to be equal to cardiac output as defined by the Starling curve



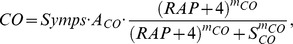
concluding our derivation of the cardiovascular model. Parameter values are given in Table 4.

**Table 4:** Parameters of cardiovascular integration in the model.

Parameter	Initial Value	Description
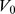	3500 ( 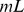 )	Unstressed blood volume
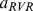	0.0296 (none)	Linear term defining effect of autoregulation on RVR
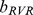	0.000839 ( 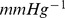 )	Constant term defining effect of autoregulation on RVR
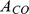	15230 ( 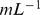 )	Maximum CO
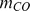	1.876 (none)	Sensitivity of the Starling curve
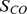	2.85 ( 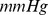 )	Set point of Starling curve
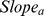	0.002408 ( 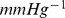 )	Linear part of compliance response to SNA
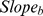	0.004543 ( 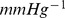 )	Constant part of compliance response to SNA

### Method of Calibration for Parameter Distributions

The goal of the calibration process is to determine a joint parameter distribution on some subset of the parameters in such a way that drawing samples from that distribution yields model responses matching those observed in human populations [5], [31]–[34]. This requires data reported from individuals to generate the prior joint distribution for the outputs, and is made more realistic by including some manner of likelihood function for each input parameter and output variable. Having no such function, we assume a uniform likelihood of all parameter sets.

The parameterization process began with the best fit parameter derived from steady state analysis (Tables 1, 2, 3, 4). An initial one-at-a-time sensitivity analysis was run to estimate the total derivative of the output function with respect to each parameter. We used Monte Carlo methods to estimate each single variable derivative, and converted into standard deviation units to normalize the comparison. The single variable derivatives were linked through vector addition, yielding a single value estimating the sensitivity 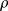 of the joint variable 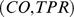 on the given parameter. We designated the critical sensitivity as 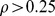 yielding 10 parameters. Those parameters deemed insensitive were left as best fit parameters through calibration. In data not shown, we lowered the threshold for sensitivity, allowing more parameters to be used in the calibration process. This resulted in similar distributions for the larger parameter set and virtually identical behavior in the model outcome.

We utilized a modified version of the Metropolis algorithm to generate and sample the sensitive parameter distribution. The Metropolis algorithm is a Markov chain Monte Carlo technique to estimate a multivariate target density by proposing new samples from the distribution and accepting them provided a probabilistic criterion is met [35]. In this variation, we sampled from the model’s space of sensitive parameters and accepted the sample based on the likelihood of its output with respect to the experimental obtained distribution. Writing 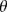 for a vector of sensitive parameter values, then 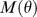 is the ordered pair at 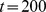 days when 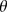 is substituted in the model for the best fit parameters. Given 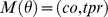, we used the smooth kernel distribution generated by the experimental data to estimate 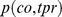 (which we write 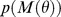), the likelihood that an individual had 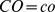 and 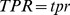. If 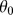 was the most recently accepted parameter vector, and 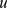 was a randomly distributed number between 0 and 1, we accepted 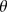 provided 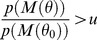 [35]. Note that if 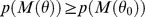 the ratio exceeds 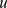. New parameter vectors are generated by allowing each component 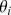 to change by adding a random number chosen from a normal distribution with mean 0 and standard deviation 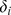 related to the expected value of the parameter. The 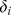 were defined to be 5% of the best-fit value for the parameter. The standard deviations were chosen so that the rejection rate was between 20% and 40% in preliminary tests of the method. The sampling process was iterated to yield a sequence of values approximating random samples from the “true” distribution. Each chain was executed 40 times: 20 to initiate the sampling sequence, and 20 retained samples. The process was restarted 15 times to ensure even coverage of the parameter space, yielding 300 individual models.

### Hemorrhage Protocol

Each sampled individual was simulated for 200 days, with steady state verified by checking that 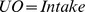. We then subjected each individual to a virtual hemorrhage by decreasing ECFV by 37.5 mL/min for 20 minutes, inducing a loss in blood volume averaging approximately 350 mL which simulated the procedure detailed in Skillman et al [5]. This process was repeated until 300 virtual patients had been generated and subjected to hemorrhage. Reseeding the algorithm reduced the possibility of strong attractors in the experimental probability distributions from unnaturally weighing the parameter distributions. Patients were classified as decompensating if their 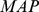 fell by more than 15 mmHg.

### Classification

We used support vector machines (SVM) to construct classifier functions 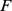 predicting model performance [10]. Given 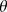 sampled from a calibrated parameter distribution, 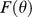 yields 1 if we predict a passing (compensating) result from the given virtual patient, and −1 otherwise. If 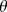 consists of a single parameter 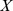, then 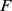 reflects the predictive power of 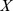 to determine compensation. In particular, if 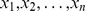 denote test values of 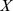, and 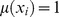 if the model compensates with 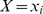 and is otherwise then 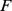 is chosen so as to maximize the sum
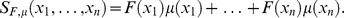



In other words, 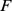 is calculated to maximize the predictive power of 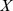 with respect to compensating/decompensating behavior in hemorrhage. We used the software svm_light, version 6.0 (http://www.svmlight.joachims.org) written by Thorsten Joachims for all classification.

The nature of SVM classification prevents easy interpretation of the classifier function. In particular, dualization of the data obfuscates the relative roles played by different parameters in establishing the outcome prediction. To get around this limitation, we used a series of SVMs to see the relative predictive powers of different combinations of parameters. This process generated a sequence of classifiers, each using a different set of parameters and variables to define a space in which classification could be attempted. The process was inductively defined: first each parameter and variable was used to construct a simple classifier. If that classifier proved more capable of predicting the outcome than the naïve classifier (every fails or everyone passes), it was retained. After examining all of the single variables and parameters, the retained parameters seeded a collection of two-parameter sets that were then used to construct classifiers. The process continued until no additional parameter could sufficiently increase the predictive power of the classifier, or until no additional parameters were available. We deemed the final result the maximally effective rubric (MER); we note that an MER is dependent on the selection of training set. The inductive procedure had the effect of reducing the number of available parameter combinations from trillions to thousands, thus making the process tractable on a desktop computer.

### Statistical Methods

Distributions were compared by Kolmogorov-Smirnov, Anderson Darling, and 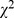 goodness of fit tests. All parameter comparisons between compensating and decompensating populations were performed as distribution fit tests. We uniformly used p<0.05 as the acceptable level of significance, and all confidence intervals were calculated at the 95% level. All statistics and random sampling were performed in Mathematica version 8 (www.wolfram.com).

## Results

### Calibration

The results of the sensitivity testing are shown in Table 5, along with the values used for the jump standard deviations 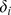. The steady state values of the 300 virtual patients were used to construct an approximation of the 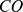 and 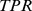 joint distribution constructed from published reports of human data [5], [31]–[34]. Individual data was collected for normotensive males, with no criterion for age. The sampled and experimental distributions were evaluated for similarity, and were not significantly different (p<0.05).

**Table 5:** Parameters used in the inclusive and sensitive tiers, along with the standard deviations of the jumps allowed in the steps of the Metropolis algorithm.0.

Parameter	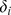
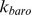	0.000035
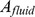	395
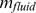	0.179
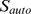	256.3
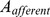	0.105
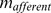	0.1815
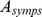	0.051
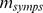	0.1745
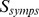	0.0265
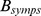	0.057

We crosschecked the population measurements of RAP and MCFP against previous observations. We found the 95% confidence interval for RAP to be [−1.74,−1.40]. In patients in cardiac failure, the 95% CI has been found to be 4–33 mmHg [36], 1–4 mmHg in normal dogs [37]. The values are statistically different (p<0.05), but are not physiologically implausible. Similarly, mean circulatory filling pressure was found to be 6.3±2.41 (SD) mmHg, as compared to 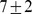 (SD) mmHg [30], [37]–[39], a difference that was not significant at p<0.05.

### Hemorrhage

The hemorrhage protocol as detailed above yielded an average blood loss of 520
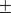
198 mL over twenty minutes. We refer to the population who suffered ≤15 mmHg drop in MAP to have compensated for the hemorrhage. Among all subjects, 43% compensated, as compared to 44% in Skillman’s experiment [5].

**Figure 2:**
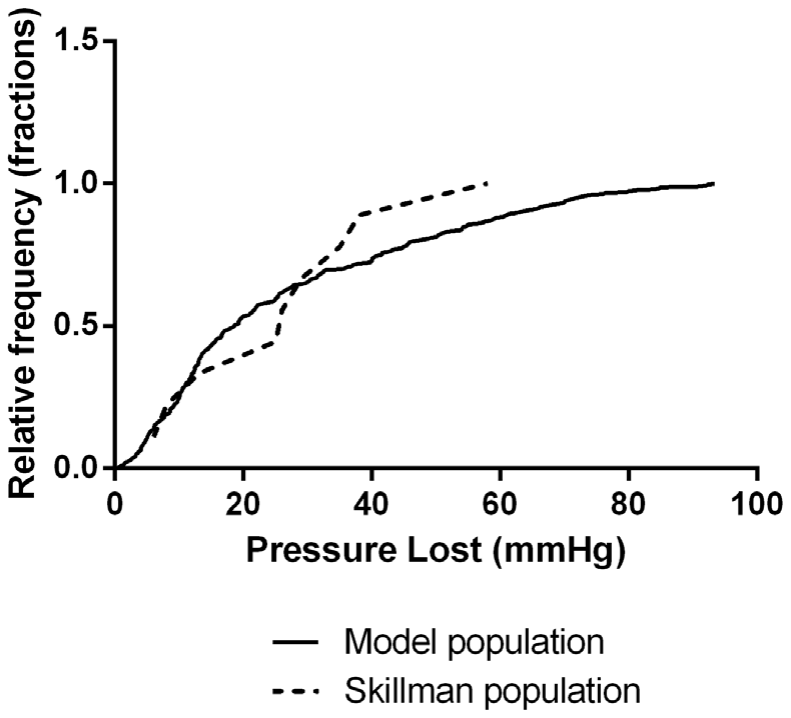
Survival function of the pressure loss experienced by individual models compared with Skillman’s data [5]; the results are similar (p<0.05). The vertical axis denotes the percentage of individuals who lost MAP less than the x-input.

**Figure 3:**
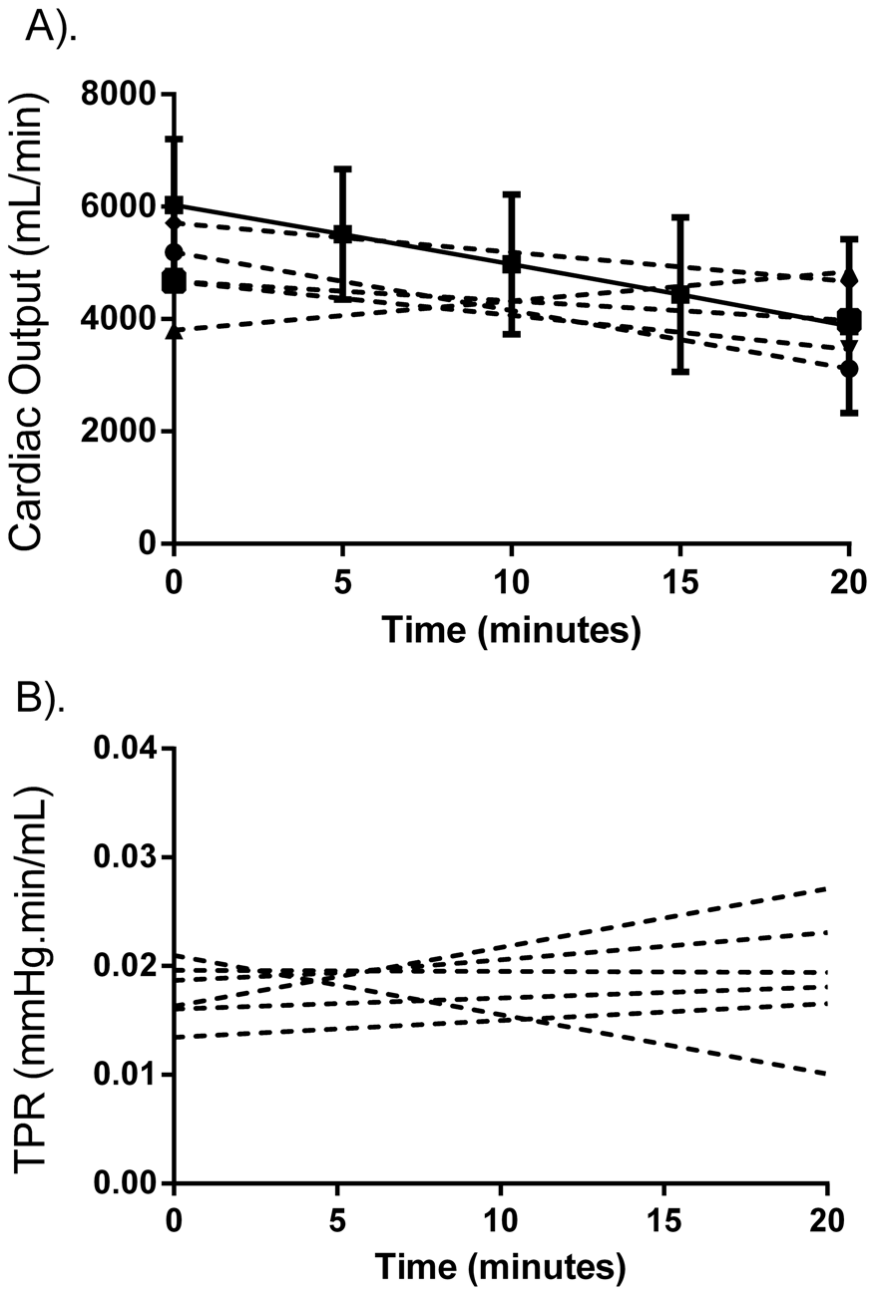
Tracking changes in CO (A) and TPR (B) during hemorrhage. The experimental data (collected at the beginning and end of the hemorrhage only) is not different from model predictions (p<0.05). The solid lines represent the mean model output, and the dotted lines are taken from Skillman’s report [5]. Error bars represent standard deviation; all experimental values are statistically similar to model output (p<0.05).

The cumulative distribution of changes in 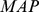 is shown in Figure 2 and is compared to the data collected by Skillman et al [5] and were not found to be significantly different from Skillman’s data (p<0.05). We also tested the 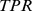 and 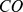 responses against Skillman’s data (reported in 5 individuals, Figure 3); the paired distributions of initial and final 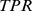 and 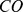 were found to be similar to those predicted by the model (p>0.05).

**Figure 4:**
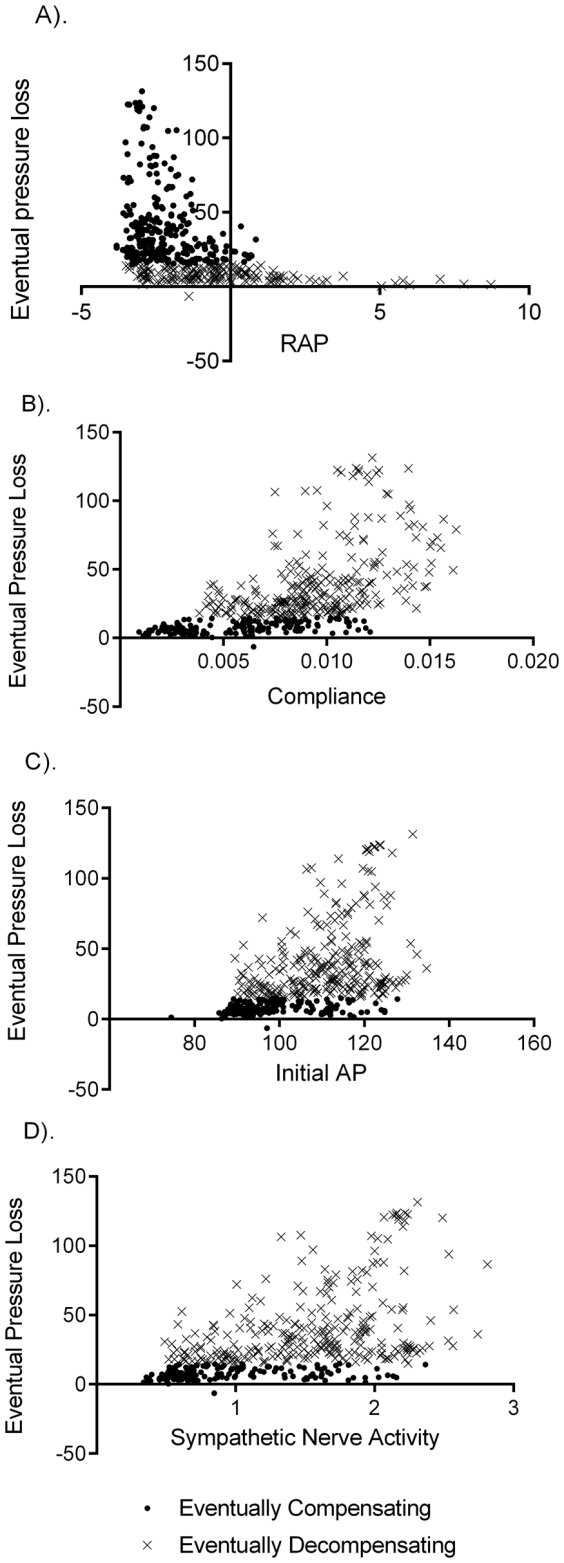
Four variables that correlate with eventual decompensation are shown. Baseline values for right atrial pressure (A) initial mean arterial pressure (C) and sympathetic nerve activity (D), and the parameter values for systemic compliance (B) were displayed correlation with compensation performance, and significant differences between compensating and decompensating populations. Solid circles represent the compensating population, and the crosses represent the decompensators. Significant overlap between the populations on the x-axis in each case illustrates the difficulties inherent in using single variables to predict eventual pressure loss.

Next we considered the predictive power of baseline variable measurements. Virtually all parameters and baseline variables showed significant differences in compensating and decompensating populations (p>0.05). The exceptions were UO, BV, and baseline afferent nerve activity. The strongest parameter correlations of model outcome were RAP, systemic compliance 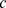, MAP, and sympathetic nerve activity (Figure 4).

**Figure 5:**
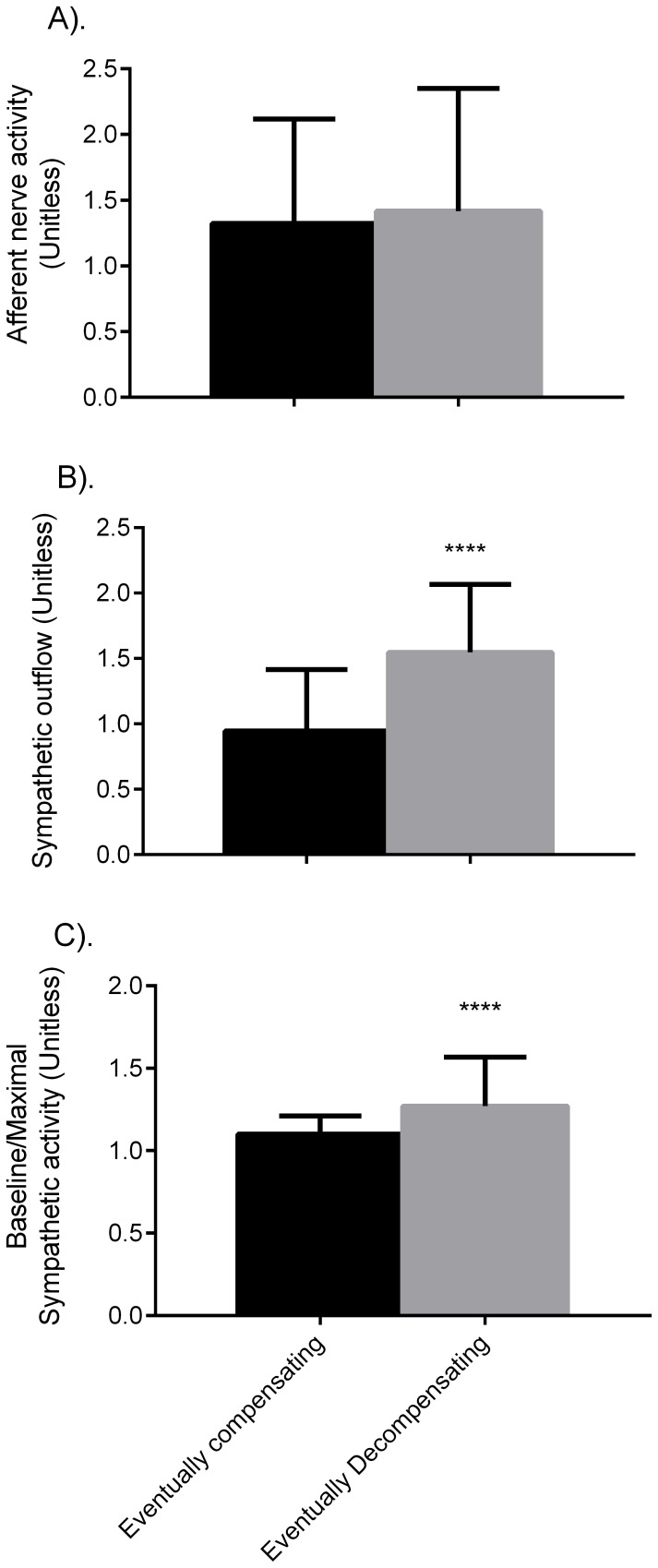
Mean nerve activities in the compensating and decompensating populations. Afferent nerve activity (A) is similar between the groups, while sympathetic outflow (B) and the ratio of baseline sympathetic outflow to maximal sympathetic outflow (C) are significantly different.

The literature suggests autonomic control differs between compensating and decompensating individuals [40], and so we analyze our populations for differences in these factors. Baseline sympathetic nerve activity and the ratio of baseline to maximal SNA are increased in decompensators (Figure 5). Considering the parameters that determine sympathetic and afferent nerve activity, the maximum and minimum sympathetic outflow and the set-point for afferent nerve activity differ between decompensating and decompensating populations (p<0.05), but all other parameters are similar.

We considered the effects of individual blood loss to performance with respect to pressure loss. Because of differences in the equations governing fluid balance, some individuals lost more blood than others. The correlation was 0.35; the immediacy of 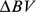 to the context of this paper invited further analysis. To test the hypothesis that the magnitude of blood loss was independently predictive of decompensation, we compared the compensating population at 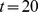 minutes, denoted 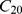, with the population that had decompensated at 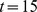 minutes, denoted 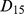. Mean 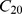 blood loss at 20 minutes was 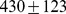 mL, compared to 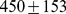 lost at 15 minutes in the 135 individuals in the 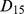 population. The differences were not significant, indicating that decompensation is more complex than simple blood loss.

**Figure 6:**
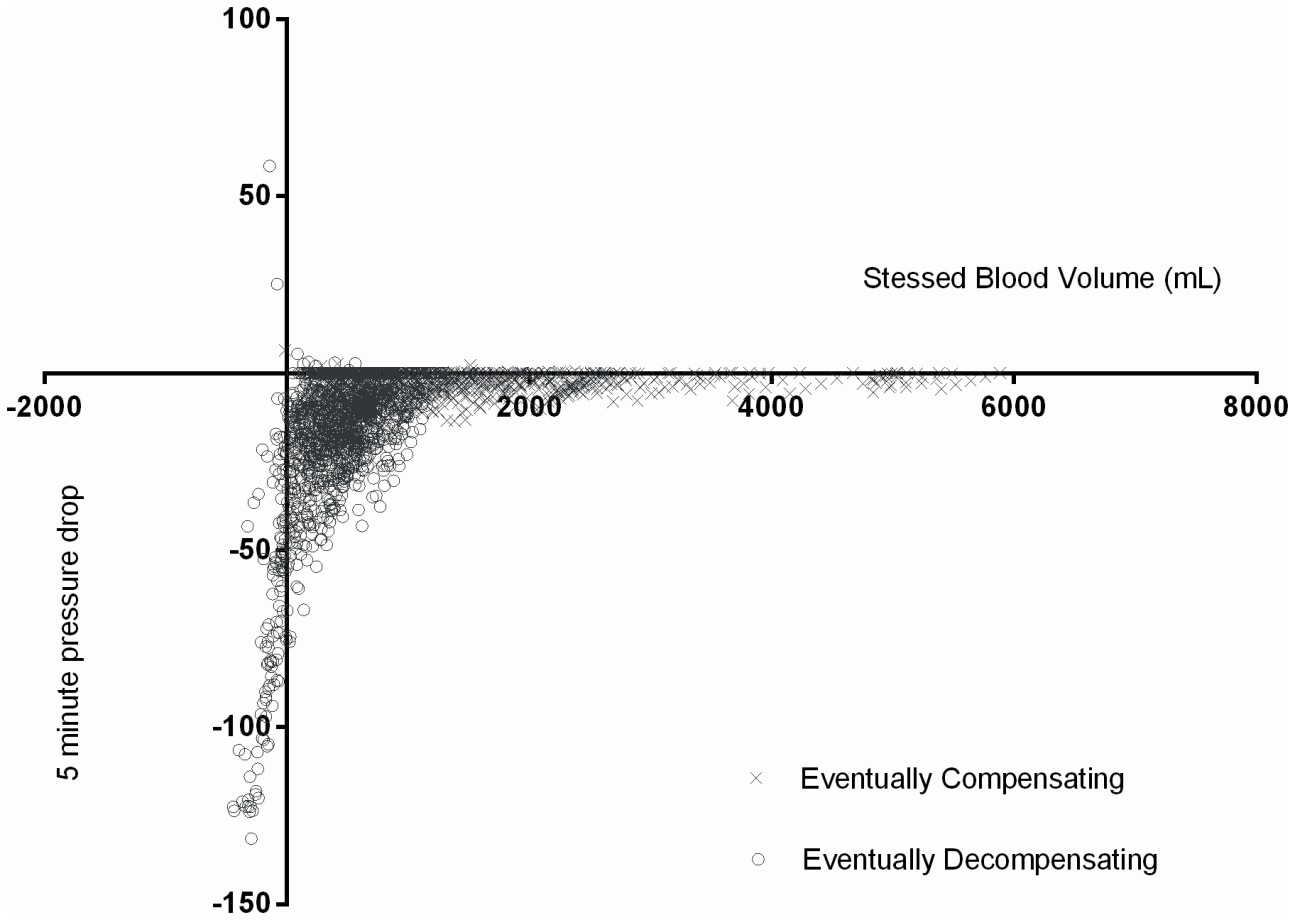
The relationship between stressed blood volume and average pressure drop over 5 minute intervals. Crosses represent eventually compensating, and circles eventually decompensating individuals. Significant pressure drops do not occur until the stressed volume approaches 0.

Finally, we considered the dynamic changes in variables as they correlated with decreases in pressure. This was not an attempt to determine causality in either direction, only correlation. The stressed blood volume presented the most striking example. Guyton defined unstressed blood volume as the volume necessary to see positive pressure develop in the circulatory tree, and the stressed volume as the complement of the unstressed volume within the total blood volume. Hence the equation defining this central component of MCFP is vital to the concept of collapse. Plotting the fall in pressure in five minute increments against the gap 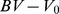 generates a good description of the point at which pressure falls precipitously (Figure 6).

### Classification

In preliminary analysis of the predictive algorithms, we found that a binomial distribution best described algorithmic performance. The power formulae for binomial distributions indicated 98 tests were necessary to determine the 95% confidence interval for predictive efficacy with a margin of error of 0.04%.

**Figure 7:**
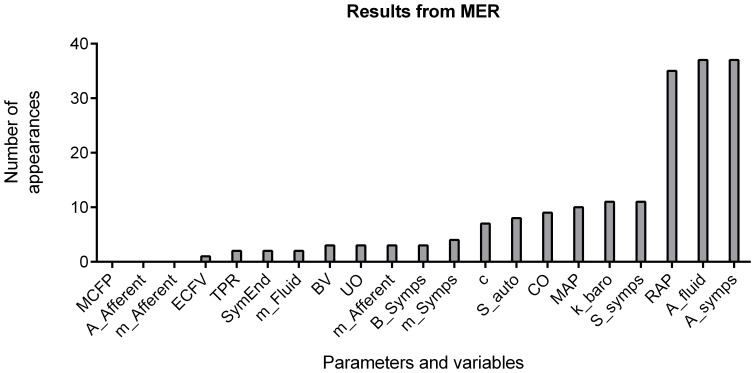
A comparison of the most influential factors predicting model decompensation. The 300 individuals were used to generate 37 collections of maximally effective rubrics (MER). The height of the bar represents the number of times the variable or parameter appeared in an MER.

We generated one hundred classifier SVMs on randomly chosen training sets drawn from the population of 300 models. We tested each classifier on the 75 individuals not included in the training set. The 95% confidence interval for overall predictive ability was [92.1%, 92.5%]. The complexity of the classifier function prevented outright analysis of individual parameter or variable contributions to decompensation. Thus, we used the MER algorithm 37 times with a cost function of 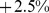 required for the addition of a new parameter or variable to generate a family of classifiers requiring fewer inputs for predictive power. Because training sets were sampled from the collected data, different sets yielded slightly different choices of parameters for constructing a classifier function. We present the most common variables seen in the classification equation in Figure 7. The MER averaged over 91% effective at predicting decompensation, but only required, on average, 5.1 variables to achieve that level of precision.

## Discussion

An individual’s parameters are different than any other individuals, and this difference extends beyond any kind of statistical uncertainty. These parametric differences inform all bodily processes, yielding subtle but real differences in the response to given stimuli. In a variety of experimental situations, system nonlinearities produce a spectrum of results that range from no effect to large effects. By smoothing the differences and relying on means as in best fit parameterization, we miss a critical part of the experimental data. By sampling calibrated parameters to generate a population of models, we can obtain a more realistic physiologic response and simultaneously increase our understanding of the relationships between interacting subsystems.

The cardiovascular response to hemorrhage exemplifies these observations. Similar individuals exposed to similar stimuli exhibit dissimilar responses, and individuals display a susceptibility to one outcome or the other. This susceptibility suggests that part of the dichotomy must be attributable to individual differences; in this paper we test this notion. This study utilizes a small integrative model with an automated parameterization algorithm. We tested the hypothesis that small integrative models can produce a spectrum of results similar to that obtained in the laboratory. When applied to the simple model presented here, the Metropolis algorithm produces a complex joint distribution of parameters that are identified, via the model, with the joint cardiac output and peripheral resistance data obtained from individual reports in the literature. To simplify the analysis, we discretized the outcome into compensating and decompensating populations in accord with the experimental standard classification. The results obtained in this study support our hypothesis. A wide range of decreases in MAP was observed in the experimental cohort, but overall the distribution was bimodal. Furthermore, the model produces single variable correlates of compensation that match previous experimental observations in lower body negative pressure (LBNP) protocols, which have been shown to be a good human model of hemorrhage [3], [41].

Three differences have been noted in LBNP intolerant individuals: 1) they have higher heart rate, sympathetic nerve activity burst frequency, and baseline central venous pressure (CVP), 2) they do not increase heart rate or SNA burst frequency in response to LBNP, and 3) they have attenuated gain in the cardiac baroreflex control of SNA [40], [42]. The third condition refers to the decreased ratio of SNA to maximal SNA observed in Figure 6. Additionally, MCFP, a correlate for CVP, was significantly different between model populations. While we do not predict heart rate in this model, increases in sympathetic outflow correlate with increased heart rate in humans. Hence the increased baseline SNA and reduced baseline to maximal SNA ratio seen in the model decompensating population would be associated with increased initial heart rate, and decreased ability to increase heart rate to aid in compensating for hypovolemia.

More generally, of the ten parameters allowed to vary, only the set point of the autoregulation responses was similar between compensating and decompensating populations. None of these parameters was seen to be independently predictive of circulatory collapse. Correlations between pressure loss and independent variables did exist for the sensitivity of the blood volume function 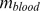 and the minimum sympathetic outflow 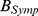. Similarly, there were significant differences between decompensating and compensating populations in most output variables, with weak correlations between pressure loss and RAP, compliance, MAP, blood volume, and baseline sympathetic outflow. Inter-variable correlation complicated any simple attempt at prediction from baseline values, despite the many correlations between value and outcome.

To better understand the influence of parameters and variables on individual compensation, we used support vector machines to generate classifying functions predicting individual behavior. Support vector machines provided, on average, 92% accurate prediction of compensation and decompensation; this average was taken over 98 partitions of the data into “training” and “testing” sets. In general, variables were more predictive than parameters. The nature of SVM classification prevented easy interpretation of the classifier function.

To circumvent this limitation, we used a series of SVMs to see the relative predictive powers of different combinations of parameters. The rubric-generating algorithm, and in particular its cost function, played an important role in decreasing the amount of information necessary for making a strong prediction. By limiting the scope of the SVM classifiers to subsets of the variables and parameters, we decomposed the classifier into its most influential parts. The algorithm described increased the tractability of the approach, reducing the search to a few hundred combinations rather than trillions of trillions. Because these rubrics were formed using a training set generated by sampling collected data, there was some variation in the particular parameters designated in this process. Common to most classifiers was compliance 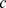, afferent nerve activity, and TPR, MCFP, and SNA. These were expected, as their covariance with the model outcome was high in both tiers. The rubric reduced the number of variables and parameters necessary to successfully classify an individual to between 5 and 6, depending on the training sample.

The value of the approach used is that, in addition to these points, we are also able to describe the range of model responses. By calibrating at steady state, the model was able to predict dynamic responses to hemorrhage that matched experimental observation with respect to single variable behavior, and to establish an etiology of decompensation that is coherent with current theories. All of these factors suggest that the model is valid, if simple. The power of this approach is that, once validated, the population can be used to make hypothetical rubrics that predict individual behavior on the basis of parameter and baseline variable values. Due to the empirical nature of this model, the particular rubrics generated are of limited value. For example, while we can suggest that baseline sympathetic nerve activity is an important indicator of circulatory decompensation, we cannot predict whether the deviant behavior is a factor of burst frequency, or mean activity, or burst amplitude. Similarly, not every variable in the model is measurable (e.g. MCFP). A more complete model would offer more opportunity for leveraging this method into useful clinical results. However, we feel that the simplicity of the model presented offers a more relatable context in which to observe and understand our methods.

In conclusion, this paper utilizes a small nonlinear model of human circulation to demonstrate a method of automated calibration from a distributed set of outputs taken from the literature. The method allows tractable sampling from complex joint distributions with minimal smoothing or data manipulation. Sampling from the distributed parameters allows one to generate a range of model patients, and to test their individual deterministic responses against a stimulus. We found strong agreement between the model chosen and the experimental outcome, and were able to use the parameter space and model outcomes to generate efficient classification functions for predicting outcomes from future patients.
